# Exploring 2D-LIPSS formation under circular polarization in ultrafast laser processing

**DOI:** 10.1515/nanoph-2025-0147

**Published:** 2025-08-28

**Authors:** Sylvain Georges, Anthony Nakhoul, Vladimir Fedorov, Paul Saby, Nicolas Faure, Hugo Bruhier, Nicolas Compère, Yoan Di Maio, Xxx Sedao, Jean-Philippe Colombier

**Affiliations:** 131842 UJM Saint-Etienne, CNRS, Laboratoire Hubert Curien UMR5516, Saint-Etienne, F-42023, France; GIE Manutech-USD, 20 rue Benoit Lauras, Saint-Etienne, F-42000, France

**Keywords:** circular polarization, laser structuring, 2D-LIPSS, self-organization, microstructured surfaces, ultrafast laser

## Abstract

The formation of isotropic two-dimensional laser-induced periodic surface structures (2D-LIPSS) under circular polarization demonstrates a unique self-organization capability. In this study, we investigate LIPSS formation under both circularly and linearly polarized light, assessing the impact of polarization dynamics, from discrete rotation angles to ultrafast changes within a single optical cycle, on pattern formation. This phenomenon stems from the surface’s ability to temporally integrate all polarization states within an optical cycle, leading to a circularly oriented response. Similarly to linear polarization, the interference between the topography-induced scattered field and the incident laser field guides the process, with feedback-driven topographical evolution sustaining structure growth. However, unlike linearly polarized light, which imposes unidirectional alignment, circular polarization promotes a more symmetric arrangement. Superposed on the hexagonal lattice of pillars with short-range order, near-field interactions generate radially oriented nanostructures that interconnect the pillars via concentric filaments. By investigating these two spatial scales separately, the respective formation mechanisms and their interplay can be clarified. Supported by electromagnetic simulations, these findings offer a comprehensive framework for understanding the mechanisms behind isotropic 2D-LIPSS formation.

## 1 Introduction

The formation of two-dimensional laser-induced periodic surface structures (2D-LIPSS) represents a transformative step in the field of laser-material interactions [1]. While traditional one-dimensional LIPSS have been extensively studied, the emergence of 2D patterns introduces new questions about the underlying symmetry, the self-organization mechanisms, and the potential functions acquired by the structured matter. What physical principles govern the spatial arrangement and morphology of these patterns? How do isotropic surface features emerge when irradiated by light that is not polarized along a single direction, and what are the broader implications of this non-directional growth? Moreover, the interaction of circularly polarized light with rough surfaces, the coherently excited circularly scattered surface waves, and their comparison to more conventional patterns formed under linear polarization, remain open areas for deeper investigation [2]. These unresolved questions challenge our fundamental understanding of ultrafast laser–material interactions and motivate exploration into new regimes of surface engineering.

Historically, LIPSS were first observed as one-dimensional patterns, aligned either parallel or perpendicular to the polarization of the laser beam, demonstrating a strong correlation with the polarization state [3], [4]. Over the years, the increasing interest in LIPSS has been fueled by their versatile applications in optics, photonics, and plasmonics. These structures offer the ability to tailor surface properties, with potential applications in plasmonic sensors, advanced photonic devices, or self-cleaning materials [4], [5], [6], [7], [8], [9], [10]. The capacity to engineer materials with periodic nanostructures opens unprecedented opportunities for nano-optics [11], heat conversion [12], as well as nanofluidics [13]. Recent reports results have also unveiled the existence of two-dimensional periodic patterns that exhibit periodicity in both directions, offering exciting possibilities for designing functionalized surfaces [14], [15]. These 2D patterns have demonstrated potential in applications such as tribology [16], [17], [18], [19], anti-reflection coatings [20], [21], and even antibacterial treatments [22].

Unlike one-dimensional LIPSS produced by linear polarization, two-dimensional structures introduce unique challenges related to symmetry-breaking, which can be addressed using crossed linearly polarized pulses in a Michelson or Mach–Zehnder setup to disrupt directionality [23], [24]. Circular polarization, with its inherent isotropic properties, is emerging as a promising method for inducing symmetric patterns [25], [26]. This approach was recognized quite early, as van Driel et al. reported that circularly polarized light produces a speckled surface with isotropic fringes [27]. However, at oblique incidence, asymmetrical patterns arise, with right- and left-circular polarization generating mirror-image diffraction patterns. At normal incidence, circularly polarized light, which lacks the directional bias of linear polarization, holds the potential to form isotropic surface patterns, an area that remains relatively underexplored in this scientific evergreen of LIPSS research. This is particularly intriguing considering that many existing explanations for LIPSS formation at low spatial frequency (LSFL) rely on surface plasmon excitation, which is inherently directed along the local polarization. It is surprising that this aspect has not been further explored to gain deeper insight into the electromagnetic feedback and hydrodynamic mechanisms that accompany these resonances. When circular polarization is used, the directionality of surface plasmon excitation is effectively averaged out, as the incident field is temporally integrated over all orientations. This raises questions about the influence of alternative mechanisms that may break the symmetry or trigger more complex surface patterns [28], such as hydrodynamic instabilities and self-organization phenomena, in particular for HSFL formation [29]. Although circular polarization can still excite plasmons [30], the strengthening of the coupling efficiency through positive feedback and directionality may differ, as the symmetry of the interaction is fundamentally altered due to the lack of alignment between the polarization of the incident wave and the surface that is undergoing structuring. These processes, which operate independently of any directional bias, could be key to understanding the formation of isotropic 2D-LIPSS [1], [5].

This article aims to address these critical questions by developing a comprehensive approach to generating and characterizing LIPSS, specifically high-spatial-frequency LIPSS (HSFL) and low-spatial-frequency LIPSS (LSFL), formed by circularly polarized light on diverse metals (iron-chromium, steel, nickel, titanium, copper, and gold). We present results on the formation of pillar-like patterns across various metals, under varying laser conditions, and using complementary imaging techniques. Additionally, we assess the formation of LIPSS under circularly and linearly polarized light, examining how slowly rotating polarization states, on the scale of a few seconds, and ultrafast rotations, on the femtosecond optical-cycle timescale, influence the pattern formation process. Through pulse-to-pulse configurations of rotating linear polarization and finite-difference time-domain (FDTD) simulations, we assess how these time-dependent variations affect the morphology, periodicity, alignment, and formation of these pillar structures.

## 2 Materials and methods

### 2.1 Material preparation

We conducted experiments on a selection of representative metals to assess their suitability for LIPSS formation, including polycrystalline stainless steel, titanium, nickel, gold, and copper. However, in this study, we focus on the material that exhibited the highest contrast in the structured patterns, coupled with a high degree of reproducibility. The main results presented here were obtained on a single crystal of iron-chromium (FeCr) oriented along the (001) direction. Single crystals are preferred to ensure optimal uniformity in the structuring process. The FeCr bar was produced by directional solidification and then cut into 10 × 10 × 10 mm^3^ using a wire saw.

All samples underwent mechanical polishing to optimize the surface condition before laser irradiation. Mechanical polishing was performed automatically using a Buehler AutoMet 250 system with successive abrasive papers of P180, P320, P600, P1200, and P2400. For single-crystal FeCr, this process was followed by diamond polishing with 3 μm and 1 μm particles.

For FeCr, an additional electrochemical polishing step was carried out after mechanical polishing using a Struers LectroPol-5 device with a stainless steel electrolyte, at 25 V for 60 s. This combination of treatments resulted in mirror-finish surfaces with a final arithmetic mean surface roughness (Ra) below 5 nm, as measured by atomic force microscopy (AFM) over a 5 × 5 μm^2^ area. Finally, the crystal orientations of the single-crystal samples were verified by X-ray diffraction prior to laser irradiation, ensuring that the cutting direction conformed to the desired crystallographic axes.

### 2.2 Laser configuration and polarization shaping

**Figure 1:**
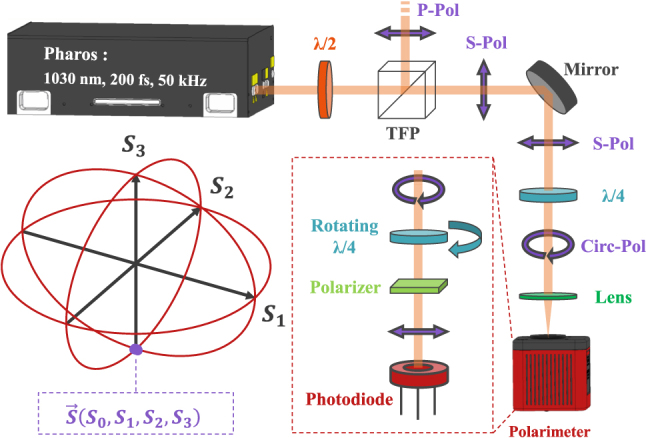
Scheme of the experimental setup showing the main elements used to control the laser power and its beam shaping. The polarimeter controlling the beam’s polarization state is positioned at the end of the optical setup. A diagram of the optics integrated within this device is provided in the figure. Finally, the Poincaré sphere is shown, providing a precise and complete visualization of the beam’s polarization state [32].

As shown in Figure 1, a femtosecond laser *Pharos* from *Light Conversion*, with a central wavelength *λ* = 1030 nm, was used for this study. For this experiment, the selected preset was fixed at a repetition rate of 50 kHz with a pulse duration of approximately 200 fs. The output power can initially be adjusted via the laser software using a variable transmission quarter-wave plate. This mechanism allows selecting a specific percentage of the laser power within a range from 400 mW to 10 W. At the laser output, a high-power variable attenuator was added. This attenuator consists of a half-wave plate and two thin-film polarizers (TFP) inclined at the Brewster angle. This setup enables a second precise adjustment of the laser output power to select the power range studied for the experiments. It also filters the initial polarization state of the laser, converting it into an *S*-linear polarization.

At the end of the optical line, after the polarization shaping stage, a focusing lens with a focal length of 250 mm is placed. The waist at the focal plane is estimated to be 39.20 μm using the *d* ^2^ method [31].

As illustrated in Figure 1, the shaping of the polarization is achieved using half-wave or quarter-wave plates placed at the end of the optical line, just before the focusing lens. These elements allow for precise control of the polarization state of the beam. The polarization state in the focal plane of the lens is monitored in real time using a polarimeter [33]. The Thorlabs polarimeter operates using a rotating quarter-wave plate followed by a polarizer, which modulates the intensity of the incoming polarized beam. Intensity modulation is measured by a photodiode, and the resulting signal is analyzed to extract the four Stokes vectors, which describe the polarization state of the beam [34].

The Stokes vectors are defined by the following relations:

(1) $$\left\{\begin{array}{c} S_{0}={\left|E_{x}\right|}^{2}+{\left|E_{y}\right|}^{2}\text{,}\\ S_{1}={\left|E_{x}\right|}^{2}-{\left|E_{y}\right|}^{2}\text{,}\\ S_{2}=2\cdot \text{Re}\left(E_{x}E_{y}^{*}\right)\text{,}\\ S_{3}=2\cdot \text{Im}\left(E_{x}E_{y}^{*}\right)\text{.} \end{array}\right.$$

The vector *S*_0_ represents the total beam intensity, which is the sum of the intensities of the transverse electric field projections *E*_*x*_ and *E*_*y*_ in the plane perpendicular to the propagation direction. The vector *S*_1_ represents the intensity difference between linearly polarized horizontal and vertical light, while *S*_2_ quantifies the intensity difference between linearly polarized light at +45° and −45°. Finally, *S*_3_ measures the intensity difference between right-hand circularly polarized (RCP) and left-hand circularly polarized (LCP) light.

Using Stokes vectors, several parameters related to the polarization ellipse can be calculated, such as ellipticity, rotation angle, and polarization contrast. Furthermore, the Stokes vectors allow the determination of the degree of total polarization (DOP), degree of linear polarization (DOLP) and degree of circular polarization (DOCP), which are defined as $\text{DOP}=100\cdot \sqrt{S_{1}^{2}+S_{2}^{2}+S_{3}^{2}}/S_{0},\;\text{DOLP}=100\cdot \sqrt{S_{1}^{2}+S_{2}^{2}}/S_{0},\;\text{DOCP}=100\cdot S_{3}S_{0}$ . These parameters enable a precise characterization of the beam’s polarization state. The most comprehensive representation of this state is achieved through the Poincaré sphere, which provides a three-dimensional visualization of the polarization ellipse as well as the direction of polarization rotation [32].

Each vertex of the sphere represents a unique polarization state, where the orientation conveys information about the ellipticity, polarization angle, and the nature of the polarization (right-hand or left-hand circular). In Figure 1, red circles connect these vertices to enhance visualization. The axes correspond to the Stokes vectors previously described in Equation (1). This real-time observation system allows for precise adjustment of the polarization state, facilitating the achievement of specific configurations such as circular polarization. Additionally, the ability to record and export polarimeter data ensures comprehensive documentation and visualization of the polarization states employed in optical structuring experiments. Lastly, during measurement, the polarimeter is aligned perpendicularly to the incident beam to ensure an accurate assessment of the polarization state as it interacts with the material.

To maintain consistency between the observation setup and experimental conditions, the sample is positioned at the focal plane under the same optical configuration. Specifically, the beam incidence on the sample is kept normal to ensure that material absorption remains independent of the angle of incidence.

### 2.3 Characterization and data processing

The surface morphology of the laser impacts was analyzed using a JEOL IT-800 SHL scanning electron microscope (SEM) equipped with a field emission gun, providing high-resolution imaging of the generated structures. The SEM imaging was conducted in secondary electron mode under high vacuum with an acceleration voltage of 10 kV. Images were captured at various magnifications in order to examine the patterns formed at different scales. In addition, a “Bruker Dimension ICON” atomic force microscope (AFM) was used to measure the topographic parameters of the surface in 3D. AFM imaging was performed in SCANASYST-AIR mode with a resolution of 2048 lines per scan, ensuring nanometric precision.

Each SEM image undergoes a standardized algorithm to extract different spatial components in the frequency domain. Initially, the Fourier spectra are computed using a conventional two-dimensional Fourier transform (FT). Since phase information is not utilized, only the power spectral density (PSD) defined as the squared modulus of the FFT spectrum, normalized to a maximum value of 1 is processed. The spectral domain is then decomposed into a {radial, angular} component space, partitioned into *N*_*R*_ × *N*_*θ*_ discrete elements, where the radial component is represented by a vector of size *N*_*R*_ × 1 and the angular component by a vector of size *N*_*θ*_ × 1. For each radial and angular coordinate, dedicated radial and angular masks are generated. The multiplication of these masks isolates specific frequency regions, defined by their radial frequency and orientation angle. Finally, the extracted features are mapped into the representation space {radial, angular}, allowing the radial PSD and angular PSD components to be obtained directly for further analysis.

### 2.4 Numerical simulation

To study the interaction of ultrashort laser pulses with rough surfaces, we numerically solve the following Maxwell equations using the FDTD method [2], [35], [36]. These equations are written as:

(2) $$\nabla \times E=-\frac{\partial B}{\partial t},\;\nabla \times H=\frac{\partial D}{\partial t},$$

where **E**(*x*, *y*, *z*, *t*) and **H**(*x*, *y*, *z*, *t*) represent the electric and magnetic field vectors, respectively. Moreover, **B** = *μ*_0_ **H**, with *μ*_0_ being the permeability of free space, and **D** = *ϵ*_0_ *ϵ*(*ω*)**E**, where *ϵ*_0_ is the permittivity of free space and *ϵ*(*ω*) is the frequency-dependent permittivity of the medium.

In our simulations, we define a computational grid with dimensions *L*_*x*_ = *L*_*y*_ = 14.6 μm and *L*_*z*_ = 1.2 μm along the *x*, *y*, and *z* directions, respectively, with spatial steps Δ*x* = Δ*y* = 10 nm and Δ*z* = 5 nm. Perfectly matched layers (PMLs) of 0.1 μm thickness are implemented at each boundary to suppress non-physical reflections. The source is modeled as a plane wave propagating along the *z*-direction. This approach allows us to reduce the computational load, especially on GPU-based calculations, while maintaining a uniform excitation over the transverse plane. As such, no transverse Gaussian profile is included in the spatial domain. The electric field amplitude is treated in relative units. While it can be rescaled to match a specific peak or average fluence, we chose not to impose a fixed fluence value, as experimental fluence typically refers to a spatially averaged or peak value across a Gaussian beam. We believe this approach keeps the simulation general and focused on the relative spatial effects of polarization rather than on exact energy coupling.

To model the incident laser beam, we used a plane wave propagating in the *z*-direction. Its temporal envelope *A*(*t*) is described by a square-sine function over a given period $A\left(t\right)=\sin^{2}\left(\frac{\pi t}{4\tau_{0}}\right)$ , where *τ*_0_ = 200 fs is the duration of the laser pulse. The electric field of the laser pulse is given by

(3) $$E\left(t\right)=\frac{A\left(t\right)}{\sqrt{1+e^{2}}}\left(\cos\left(\omega_{0}t\right)\;u_{x}+e\sin\left(\omega_{0}t\right)\;u_{y}\right),$$

where *e* represents the ellipticity of the polarization, which controls the relative amplitude of the *E*_*x*_(*t*) and *E*_*y*_(*t*) components. This modifies the orientation of the electric field vector in the *xy*-plane. When *e* = 0, the polarization is linear; for *e* = 1, it becomes circular, and between these two values all intermediate ellipses are possible. The sign of *e* determines the direction of the polarization rotation (clockwise or counterclockwise).

After defining the components *E*_*x*_(*t*) and *E*_*y*_(*t*), we apply a rotation in the *xy*-plane using a 2D rotation matrix to orient the electric field vector at a given azimuthal angle *θ*. This step is crucial in analyzing the effect of polarization on the light–matter interaction with rough surfaces. In this study, we used both methods to simulate surface roughness.

To model surface roughness, two complementary approaches are considered, depending on whether we aim to generate synthetic roughness profiles or reproduce experimentally observed textures. The first approach is based on the random roughness function *R*(*x*, *y*), which generates height variations relative to a reference plane. This roughness is described by a spatial correlation function accounting for inhomogeneities. Although we compared surfaces with root-mean-square (RMS) height *σ* = 5 nm, close to experimental surface states, and *σ* = 50 nm, we observed that for a single pulse, only the contrast of the absorbed energy pattern is affected. Therefore, *σ* = 50 nm RMS was selected to facilitate the visualization of results. The correlation length is set to *ξ* = 100 nm, corresponding to sub-wavelength inhomogeneities necessary for the formation of high-frequency periodic structures. The spatial coherence of these inhomogeneities follows a Gaussian correlation function:

(4) $$C\left(X,Y\right)=\sigma^{2}\exp\left(-\frac{X^{2}+Y^{2}}{\xi^{2}}\right).$$

The second approach consists in using SEM images of experimentally textured surfaces, which are converted into digital height maps to reproduce realistic topographies. In this case, we take into account the peak-to-peak height of the structures, measured by AFM as *h* = 150 nm. These two types of surface topographies are then incorporated into FDTD simulations to study the effect of surface irregularities on the light–matter interaction. This dual approach allows us to compare the influence of controlled statistical roughness and realistic surface features on the laser-induced structuring process.

Furthermore, we calculate the energy *Q*(*x*, *y*) absorbed by the surface, obtained by integrating the energy per unit volume over time along the longitudinal direction, through the simulated local electric field **E**(*x*, *y*, *z*, *t*). This energy is expressed as:

(5) $$Q\left(x,y\right)=2\epsilon_{0}n^{\prime }n^{\prime \prime }\omega_{0}\overset{\;\infty }{\underset{-\infty }{\iint }}{\left|E\left(x,y,z,t\right)\right|}^{2}\;dt\;dz,$$

where *n*′ and *n*″ represent the real and imaginary parts of the complex refractive index of the absorbing medium. This approach establishes a direct link between the simulated electric field polarization and the energy actually absorbed by the inhomogeneous surface. It is essential for predicting the periodic energy distribution that ultimately forms topography patterns to evaluate the efficiency of various laser pulse configurations.

## 3 Results

### 3.1 Structuring with circular polarization

The formation of 2D-LIPSS with circular polarization is examined by analyzing the resulting surface morphologies for under different laser conditions. Variations in periodicity, structural arrangement, and feature dimensions are investigated through AFM and SEM, complemented by post-processing treatments in the spectral domain, providing a detailed assessment of how a circularly polarized femtosecond laser beam influences the development of these patterns.

The circular polarization contrast was controlled using the Stokes vectors described by Equation (1), enabling a DOCP greater than 99 % to be extracted. To obtain isotropic structuring, a DOCP greater than 95 % is essential. Below this threshold, the periodic structures obtained have a preferential axis. This characterization ensures precise control of the polarization state of the beam before interacting with the surface, ensuring the reproducibility of the structures obtained.

**Figure 2:**
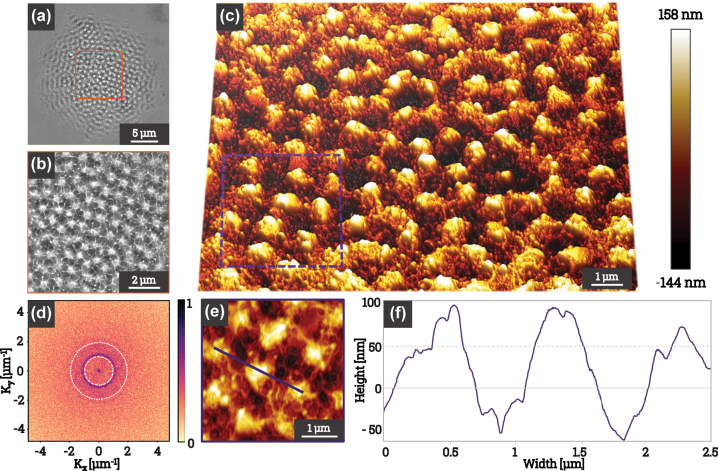
Topographic characterization of 2D-LIPSS. (a) SEM image of structures formed on a FeCr(001) surface under 50 fs laser pulses with circular polarization (DOCP > 99 %) at a peak fluence of 0.14 J/cm^2^. (b) Higher-magnification SEM image corresponding to the orange-marked region in (a), highlighting periodic HSFL and LSFL structures. (c) AFM image of the structured area, revealing an average feature height of approximately 150 nm. (d) Fourier transform of the SEM image in (a), with white dashed circles indicating the characteristic wavelength and half-wavelength. (e) High-resolution AFM scan from (c), with an extracted cross-sectional profile. (f) Topographic chart of the cross-section extracted from (e), showing height variations.

The microstructural features formed under circularly polarized femtosecond laser pulses are presented in Figure 2. The SEM image in Figure 2(a) reveals a well-organized pattern of periodic structures across the irradiated surface. A higher-magnification view, presented in Figure 2(b), highlights the coexistence of both HSFL and LSFL at the center of the laser impact. The LSFL structures appear as pillar-like patterns with diameters close to half the laser wavelength ( ∼500 nm), forming a pseudo-periodic arrangement with an average spacing of approximately 0.9 µm. In contrast, the HSFL structures manifest as radial filaments that interconnect the bumps with their nearest neighbors, adding to the overall complexity of the pattern.

The AFM measurements, presented in Figure 2(c), reveal a three-dimensional topography with a peak-to-peak structure height of approximately 150 nm. The FT analysis of the SEM image, shown in Figure 2(d), confirms the periodic nature of the LSFL, with distinct frequency components corresponding to the laser wavelength and half-wavelength, as indicated by the white dashed circles. The LSFL periodicity is estimated at approximately 910 nm. In contrast, while the HSFL features are distinguishable at the periphery of the FT spectrum, their lower contrast and dilution in frequency space limit accurate period determination. A localized AFM scan, shown in Figure 2(e), along with the corresponding cross-sectional profile in Figure 2(f), further highlights the height variations and morphological characteristics of the structured region.

Understanding the mechanisms of formation of these structures requires a comparative analysis of their development under circular and rotated linear polarizations. The following section explores how polarization, both slow and ultrafast rotational, influences the morphology and periodicity of LIPSS. By comparing the polarization states and pulse conditions, we investigated how these factors shape the structural evolution of the irradiated surface. Furthermore, beyond polarization effects, material properties also play a role in structure formation. A detailed analysis of the response of different metals to a circularly polarized laser beam is presented at the end of this paper, providing further insights into the interplay between optical coupling and material-dependent transport characteristics.

### 3.2 Comparative analysis of circular and linear polarization in 2D-LIPSS

To conduct a comprehensive comparative study on surface structuring under different polarization and energy density conditions, a rigorous experimental approach was adopted. This method focuses on a systematic analysis of the effects related to circular and linear polarization, as well as the variation in the number of laser pulses applied to the sample.

Initially, laser irradiation was conducted using circular polarization. A quarter-wave plate was used to obtain high-contrast circular polarization (DOCP higher than 99 %). This circular polarization was then used to generate impacts on the sample with varying numbers of pulses. During this set of the experiments, the successive laser pulses were spaced 2 s apart in time, allowing the material to return to its relaxation state after each pulse. The laser fluence was controlled and maintained constant in each configuration, with the power being verified at every step.

We carried out a second set of laser irradiation experiments in a similar fashion, with linearly polarized laser pulses. For this, the quarter-wave plate was replaced by a half-wave plate, enabling control over both the polarization orientation and the DOLP, which was found to be 97 % ± 1 %, depending on the orientation angle. As with the circular polarization, impacts were made with varying numbers of pulses. However, in this case, the polarization angle was modified after each pulse. The variation in the polarization rotation angle step depended on the total number of pulses *N*, following the relation as Δ*θ* = 2*π*/*N*. Thus, the dose, based on the sum of the average fluence of each pulse, can be written as $D_{N}^{\text{lin}}=\sum_{n=1}^{N}f_{m}\left(\theta_{n}\right)$ , where *f*_*m*_ is the average fluence per pulse, and the polarization angle *θ*_*n*_ for the *n*-th pulse is defined by *θ*_*n*_ = (*n* − 1)Δ*θ*.

In addition to these configurations, a variant was also implemented in which a *π*/2 jump in polarization was applied between every two consecutive pulses. This approach aimed to break the symmetry between successive pulses, introducing a larger angular modulation. This variant was systematically applied for all configurations to study the impact of strong polarization discontinuities on the resulting surface structures.

To evaluate the repeatability and robustness of the observations, this series of experiments was repeated multiple times for each configuration, allowing a reliable analysis of the surface structuring phenomena under varying polarization dynamics and energy density conditions.

**Figure 3:**
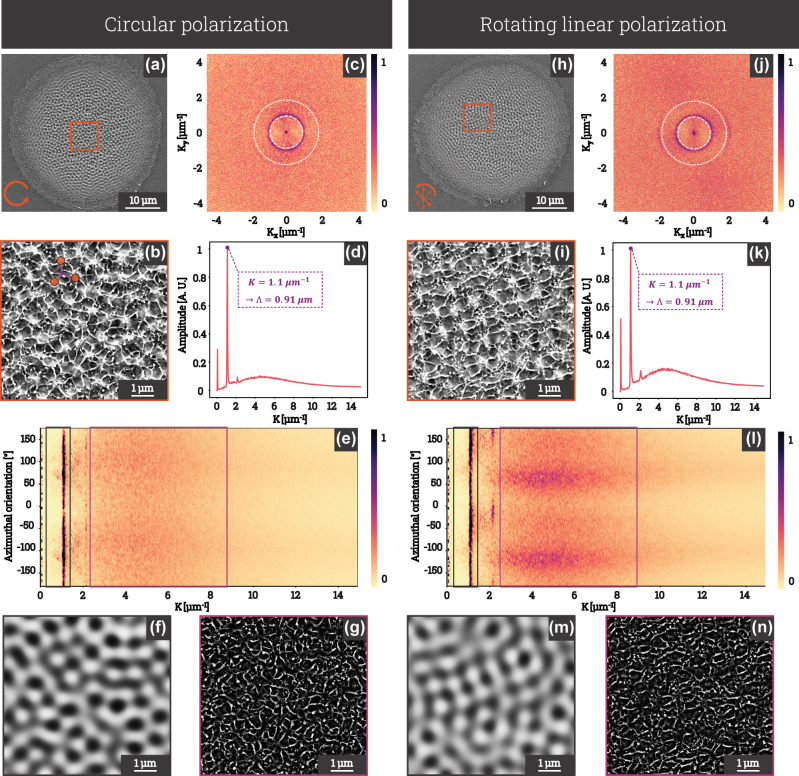
SEM images of 36 fs laser impacts on an FeCr(001) sample, generated with an average fluence of 0.145 J/cm^2^ and a pulse duration of 225 fs: (a) impact with circular polarization (DOCP > 99 %, indicated by a circular arrow symbol) and (h) impact with rotating linear polarization (DOLP = 97 % ± 1 %, indicated by linear arrows tilted at specific angles, with transparency illustrating that pulses are spaced by several seconds), with a variable azimuthal angle. (b, i) Higher-magnification views of the impacts, corresponding to the regions highlighted by orange squares in (a) and (h), respectively. (c, j) Fourier transforms (FT) of the structures within the impacts for circular and rotating linear polarization. (d, k) Radial and (e, l) angular power spectral densities (PSD) for circular and rotating linear polarization. (f, g) and (m, n) show spectrally filtered images of (b) and (i), FT inverse of frequencies within the grey and purple boxes of (e) and (l).

Figure 3 shows SEM images of FeCr(001) surfaces patterned by 36 fs laser pulses under different polarization conditions. Panels (a) and (h) show the resulting surface morphologies for circular and rotating linear polarization, respectively, the latter involving a progressive azimuthal rotation of the polarization axis. The higher magnification views of around ×6.6 in (b) and (i) give a detailed look at the structural features within the laser impact zones. In (b), a region highlights a trihedral junction where optical scattering from three LSFL domains interferes; HSFL filaments are observed to radiate toward the center, emphasizing the combined influence of the polarization state and near-field coupling induced by local surface roughness. To further analyze these structures, (c) and (j) show Fourier transforms of the patterned regions, revealing distinct spatial frequency distributions for each polarization state. Next, the periodic features of the patterns are quantified by radial and angular power spectral density analyses, presented in (e)–(f) for circular polarization and (k)–(l) for linear rotational polarization. Finally, Figure 3(f), (g), (m), and (n) highlight the morphological differences by filtering the frequencies within the gray boxes for (f and m) and the purple boxes for (g and n). In this way, the morphological comparison of HSFLs and LSFLs in the two polarization cases can be made visually.

Under circular polarization (Figure 3(a)), the surface exhibits periodic structures similar to those observed on titanium [37], cobalt [38], and stainless steel [39]. The corresponding Fourier transform (Figure 3(c)) reveals an annular distribution of spatial frequencies, characteristic of LSFL with a periodicity of approximately 910 nm. These structures arise from the interference of diffracted waves with the incident beam during the laser interaction [40], [41]. In contrast to LSFL, no distinct periodicity is observed for HSFL, as evidenced by the homogeneous appearance of the Fourier transform at high frequencies. HSFL manifest as filaments connecting the closest surface irregularities, rather than forming cross-linked networks. This configuration leads to an isotropic distribution of structures, preventing the identification of any preferential direction.

Under rotating linear polarization (Figure 3(h)), the structured surface morphology exhibits significant differences compared to the circular polarization case. While the observation of LSFL reveals a network distribution that appears similar to that observed under circular polarization, HSFL exhibit a preferred orientation. The progressive azimuthal variation of the polarization introduces anisotropic effects, observable in the high-magnification SEM image (Figure 3(i)). The Fourier transform (Figure 3(j)) displays an annular distribution for LSFL, indicating the presence of a globally isotropic bump network. However, the preferred orientation of HSFL is clearly evident through the presence of oriented clusters at high frequencies in the Fourier transform.

Furthermore, the analysis of the radial power spectral density provides a more detailed examination of the characteristic frequencies (Figure 3(d)), revealing several distinct peaks. The first low-frequency peak corresponds to large-scale structures, while a sharp second peak, estimated at 0.91 μm of period, confirms the periodicity of LSFL. A subtle peak around 2.4 μm^−1^ is attributed to the second harmonic of the LSFL structure, due to its well-defined period but non-sinusoidal shape. Additionally, the broad peak between 2.5 and 8 μm^−1^ is associated with HSFL structures, though their contribution is weaker and appears diluted into the spectral distribution. Upon comparison with Figure 3(k), the same sharp peaks are observed under rotating linear polarization, indicating that the periodicity of LSFL remains consistent irrespective of polarization type. However, the primary difference lies in the contrast between the LSFL peak and the HSFL bump. Specifically, the ratio between the contributions of HSFL and LSFL appears higher under rotating linear polarization than under circular polarization, indicating a more pronounced influence of high-frequency structuring in this configuration.

In Figure 3(e), the structures oriented along azimuthal directions at 60° and 120° are clearly visible for 1 < *K* < 2 μm^−1^, with a slight angular dispersion. These structures are characteristic of a three-order symmetry, indicative of hexagonal lattices. This corresponds to a highly compact pattern that naturally emerges when the physical cause is isotropic and instabilities lead to a locally organized state that propagates optimally. Hexagonal structures are commonly observed in various self-organizing systems, as they represent a stable and optimized solution under physical or chemical constraints (thermo-convection, Turing patterns, or hydrodynamic instabilities), resulting from a competition between energy minimization, opposing force interactions, and mode saturation. Although not universal, it is noteworthy that these structures form more efficiently and frequently under circular polarization compared to rotating linear polarization. For the latter, structures oriented at approximately ±90° within the range 1 < *K* < 2 μm^−1^ can be observed in the azimuthal orientation of Figure 3(l). These correspond to two-order symmetric structures, which are more challenging to observe due to their localized nature, rotating across the entire spot. Additionally, boundary conditions at the impact edges tend to distort the regularity of these structures.

The spectrally filtered images shown in Figure 3(f), (g), (m), and (n) enable a more precise morphological comparison of LSFL and HSFL under circular and linear polarization, by filtering the frequencies associated with LSFL (gray box) and HSFL (purple box), respectively.

The filtered image in Figure 3(f) reveals localized hexagonal structures that are not clearly visible in Figure 3(e), due to the lack of uniformity of these features across the entire spot surface. Nevertheless, Figure 3(e) shows weaker peaks around 2.2 μm^−1^, spaced by 60°, which are characteristic of the FFT signature of a hexagonal structure. The low amplitude and poor definition of these peaks are attributed to their origin as a weak second harmonic of the LSFL structures visible in the SEM image. In Figure 3(l), two distinct peaks at 2.2 μm^−1^, separated by 180°, indicate the linear orientation of the LSFL structures in the case of linear polarization. The frequency range between 2.5 and 9 μm^−1^, corresponding to the HSFL regime, exhibits two broad angular peaks spaced by 180°, oriented 90° relative to the LSFL structures. These broad peaks indicate that the HSFL structures have a preferential linear orientation, despite their low spatial uniformity. This characteristic is completely absent in Figure 3(e), corresponding to the circular polarization case. Furthermore, images (g) and (n) highlight the morphological differences between high-frequency structures depending on polarization, with HSFL under circular polarization displaying a nearly isotropic distribution, although a slight orientation can still be observed. A possible explanation for the observed orientation of these structures, despite the use of circular polarization, is that feedback from the initial surface morphology may have influenced the near-field distribution and contributed to symmetry breaking in the HSFL formation process. While under linear polarization, the HSFL are preferentially aligned along specific directions. Spectral analysis confirms that, although the periodicity of LSFL remains largely constant, the progressive rotation of linear polarization imposes alignment constraints that influence the dynamics of high-frequency structure formation.

Moreover, when a variant with *π*/2 polarization jumps every two pulses is combined with a gradual rotation (i.e. $0,\frac{\pi }{2},\theta_{n},\theta_{n}+\frac{\pi }{2},\theta_{n+1},\theta_{n+1}+\frac{\pi }{2},\ldots \;$ ), the LIPSS structures are similar to those shown in Figure 3(i). However, due to the different sequence of polarization orientations, the final pulse has a distinct linear polarization orientation compared to that with continuously varying polarization. As a result, the HSFL which are more sensitive to the final pulse, adopt a preferential orientation different from that observed in Figure 3(i). Counterintuitively, this observation suggests that the order of the polarization sequence has minimal impact on the degree of anisotropy of the nanostructured surface.

### 3.3 Electromagnetic simulations

This section presents the results obtained using the simulation code described earlier in Section 2.4. The objective of these simulations is to reproduce, using the FDTD method, the different experimental configurations explored. These configurations include beam shaping with circular polarization and linear polarization at adjustable angles. The orientation of the circular polarization angle or the determination of the circular polarization direction is achieved using Equation (3), by defining a parameter *e* equal to +1 for right circular polarization and −1 for left circular polarization. This approach enables a detailed analysis of the interaction between the electromagnetic field and the surface under experimental conditions.

**Figure 4:**
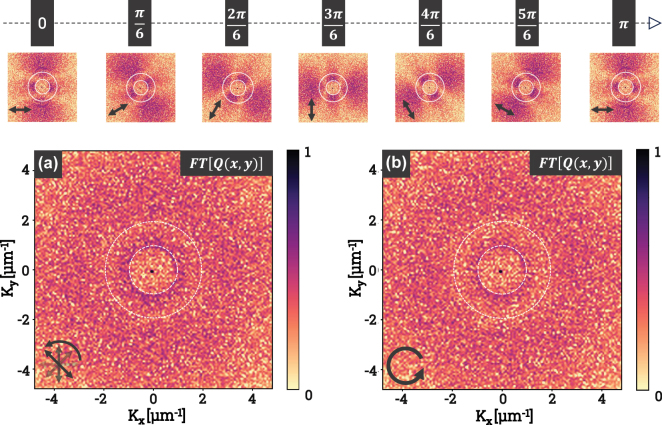
The top row presents the Fourier transforms (FTs) of the energy absorbed by linearly polarized pulses, with a polarization angle varying from 0 to *π*, in steps of *π*/6. (a) Shows the FT of the sum of all the energies absorbed by 36 linearly polarized pulses with variable angles from 0° to 350°. (b) Is the FT of the sum of the energies absorbed by 36 circularly polarized pulses. The results shown in this figure are obtained using the finite-difference time-domain (FDTD) numerical simulation method.

Figure 4 shows the modeling results obtained. The top panel of the figure shows a subset of the 36 FTs generated by the simulation code, including 7 FTs corresponding to polarization angles ranging from 0 to *π*, in increments of *π*/6. The orientation of each linear polarization was calculated using Equation (3), while the absorbed energy *Q*(*x*, *y*) was computed using Equation (5). This representation enables the identification of spatial frequency distributions associated with LSFL and HSFL, which are oriented parallel and perpendicular to the linear polarization, respectively. These periodic structures depend on the polarization state, with each configuration imprinting a preferred energy absorption pattern on the material.

For each polarization angle, the simulation was performed starting from a pristine surface generated using Equation (4), with root-mean-square height parameters *σ* = 50 nm and correlation length *ξ* = 100 nm. These parameters describe surfaces generated using the roughness function *R*(*x*, *y*), representing sub-wavelength inhomogeneities necessary for the appearance of HSFL structures. The case studied here is intentionally simplified, assuming no feedback between successive pulses. Additionally, simulations were also carried out with a higher RMS height of *σ* = 5 nm. The difference between the two lies in the contrast, but the overall result remains unchanged.

The sum of linear polarizations is achieved by combining the contributions from linear polarizations, each separated by an angle of 10°. This approach reduces the influence of successive orientations and generates a homogeneous response in the (*x*, *y*) plane. Figure 4(a) illustrates the normalized sum of the 36 FTs obtained for varying linear polarization angles. In this figure, the clusters corresponding to LSFL (central ring) and HSFL (periphery) exhibit homogeneous structures with well-defined periods. The spectral density is maximized for LSFL between 0.8 μm^−1^ and 1.2 μm^−1^, and for HSFL between 2.5 μm^−1^ and 3.5 μm^−1^.

By comparison, Figure 4(b) shows the results obtained using counterclockwise circular polarization. The resulting FTs reveal no significant differences compared to those obtained from the sum of linear polarizations. In both cases, the LSFL and HSFL structures are clearly discernible. However, the marked similarity between the FTs obtained from these two approaches contrasts with previously reported experimental results.

This discrepancy may be attributed to the lack of feedback from the absorbed energy of previous pulses in the simulation. In experimental conditions, the surface interacts successively with each pulse, leading to successive variations in topographic properties induced by the absorbed energy. This feedback mechanism, absent in our simulations, is believed to primarily explain the differences observed between the simulated and experimental results. For LSFL formation, feedback is generally driven by local ablation in regions of maximal field intensity. A common approach to account for this feedback involves iteratively removing material where the local intensity exceeds a threshold after each pulse, as demonstrated by ref. [42]. However, for HSFL, the situation is more complex due to the involvement of transient molten flow and convective instabilities, which require coupling electromagnetic, thermal, and hydrodynamic solvers. Implementing these effects presents a significant computational challenge, particularly in 3D and at high spatial resolution, but is crucial for accurately modeling the final surface morphology. We acknowledge this limitation and have expanded the discussion in the manuscript to address these complexities.

To gain a deeper understanding of the absorption mechanisms during the structuring process, where 36 laser pulses are used, we simulate the optical coupling of an additional pulse polarized on experimentally obtained scanning electron microscopy images.

**Figure 5:**
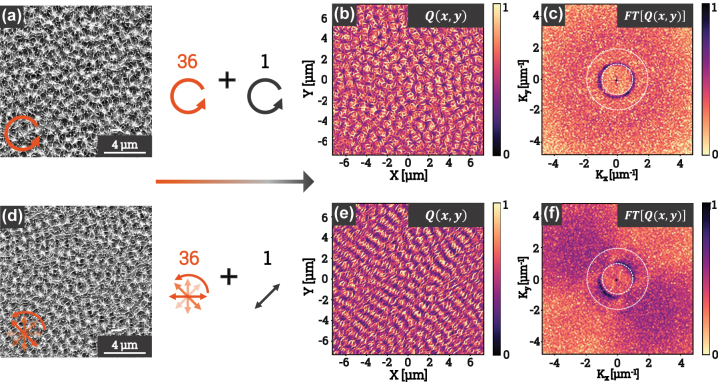
SEM images of the structures generated by 36 circularly polarized pulses (a) and rotating linearly polarized pulses (d). (b) and (e) Show the energy absorbed by the interaction of the surface observed in images (a) and (d) with a simulated pulse of circular polarization (b) or linear polarization (e). Finally, (c) and (f) present the Fourier transforms of the absorbed energy distributions from (b) and (e), providing insight into the spatial frequency characteristics of the structured regions.

Figure 5(a) presents the SEM image of a surface structured by 36 circularly polarized pulses. This image reveals the periodic structures characteristic of circular polarization. It is used as the initial surface in the simulation, with a height *h* = 150 nm. A 37th pulse with circular polarization is then simulated to compute the absorbed energy resulting from the interaction between the pulse and the surface. The absorbed energy distribution and its Fourier transform (FT) are shown in Figure 5(b) and (c), respectively. The absorbed energy is isotropic, with regions of higher intensity explained by the initial surface state. The corresponding FT highlights both LSFL and HSFL clusters, which are characteristic of circularly polarized beams.

Similarly, the SEM image of a surface structured by 36 pulses with linearly polarized beams at varying angles (Figure 5(d)) undergoes the same treatment. This image is used as the initial surface, and a 37th linearly polarized pulse, inclined by 10° relative to the angle of the 36th pulse, is simulated. The absorbed energy distribution for this configuration is shown in Figure 5(e), displaying a preferred orientation influenced by both the initial surface state and the polarization angle of the final pulse. The presence of a more asymmetric distribution of HSFL in the results obtained from the 36 pulses of linearly polarized beams with varying angles can thus be attributed to the influence of the final pulse. Indeed, Figure 5(e) reveals that HSFL formation appears to be significantly influenced by the absorbed energy from the near-field of the last pulse, which dictates the orientation of the nanoscale structures. The FT of the absorbed energy, presented in Figure 5(f), exhibits features characteristic of LIPSS (LSFL and HSFL) formed under linearly polarized beams.

## 4 Discussion

### 4.1 Material effect

**Figure 6:**
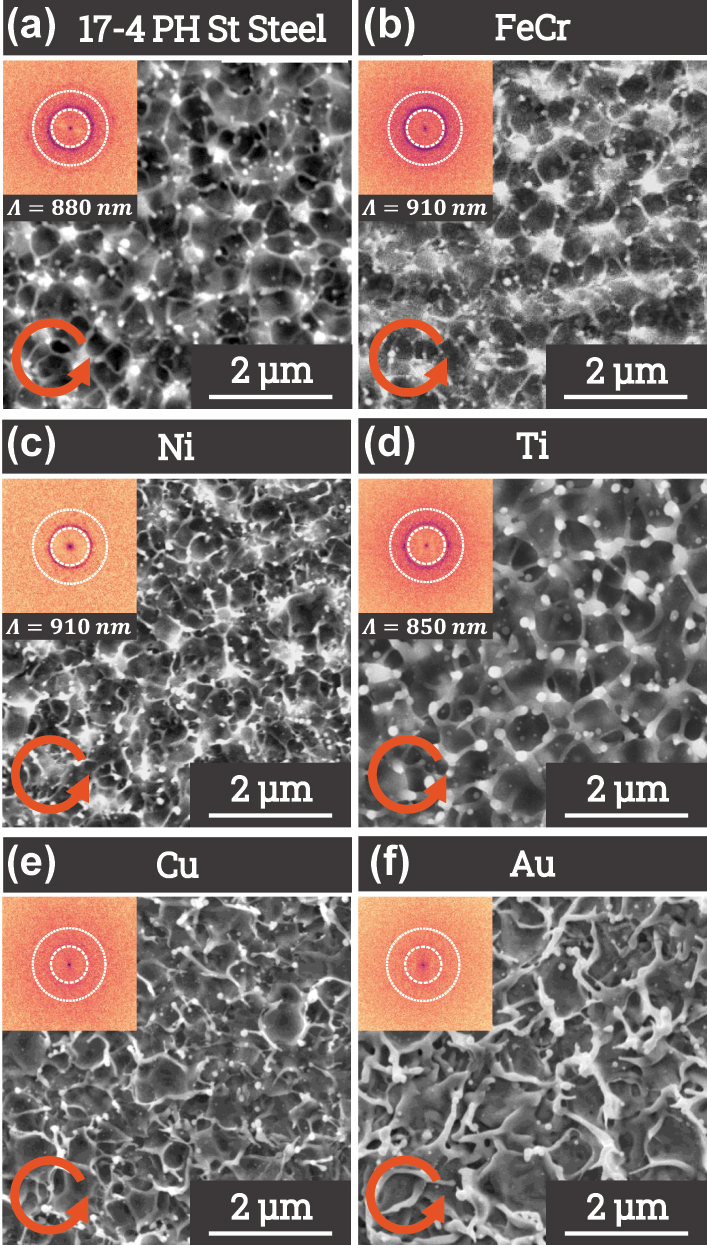
SEM images of femtosecond laser beam impacts with circular polarization (*λ* = 1030 nm) on various materials: (a) 17-4 PH stainless steel (*f*_*m*_ = 0.18 J cm^−2^, 50 pulses), (b) iron-chromium alloy (*f*_*m*_ = 0.18 J cm^−2^, 50 pulses), (c) nickel (*f*_*m*_ = 0.26 J cm^−2^, 50 pulses), and (d) titanium (*f*_*m*_ = 0.18 J cm^−2^, 50 pulses), (e) copper (*f*_*m*_ = 0.75 J cm^−2^, 50 pulses), and (f) gold (*f*_*m*_ = 0.75 J cm^−2^, 50 pulses). Each image is accompanied by its Fourier transform and the period of the pattern generated, displayed in the top-left inset.

A wide range of fluences and pulse numbers was tested on six different materials to investigate the formation of structures induced by a circularly polarized femtosecond laser beam. Figure 6 presents the results for each material, with the pulse number fixed at 50. The formation of LIPSS under linear polarization is well-documented for metals such as Ni, Ti, Cu, and Au [43], [44], [45], and is therefore not detailed here.

Structures are observed on all tested materials, though the fluence range and pulse number required for their formation vary depending on the material. These parameters are influenced by the materials’ transport properties, particularly electronic thermal conductivity, which governs the confinement length according to the two-temperature model [46], [47]. The enhanced thermal diffusion is stopped by the electron–phonon relaxation following laser interaction, as the energy exchange rates dictate the extent of thermally affected depth and subsequent molten layer thickness [44], [47]. A shorter relaxation time restricts electronic heat conduction, creating steep energy gradients that enhance the contrast of surface structures. Additionally, the fluence required for pattern formation is indirectly influenced by the material’s electronic and thermal properties.

For metals such as 17-4 PH stainless steel, nickel, titanium, and iron-chrome, each exhibiting similar thermal conductivities (*K* < 100 W m^−1^ K^−1^) corresponding to high electron-phonon collisional rates, structure formation occurs over a broad range of fluences and pulse numbers. This suggests that for these metals, the estimated length of the energy gradient within the molten layer remains within a comparable range. Due to this similarity of thermal transport, 2D-LIPSS form on these metals at a comparable average fluence of 0.18 J/cm^2^. They also display a similar contrast in their laser-induced topographical response.

On the other hand, for metals such as copper and gold, which have significantly lower electron–phonon coupling [48], [49] and higher equilibrium thermal conductivities (*K* > 300 W m^−1^ K^−1^), the parameter range for structure formation is considerably narrower. The amplitude of the generated structures is lower, and the features in the FT are significantly less distinct. Consequently, no two-dimensional periodic structures were observed on these materials within the fluence range tested. This limitation is likely due to the less efficient coupling between electrons and phonons, which sustains electronic energy diffusion in depth and inhibits the formation of periodic 2D patterns. Finally, the reduced structure amplitude and low contrast in the FT further suggest that periodicity is either weakly defined or entirely absent in these materials.

The FTs of the impact topography allow identification of the period of the generated structures and provide a qualitative estimate of their amplitude. Notably, the structures observed on different materials exhibit a similar period, suggesting that periodicity is not dictated by thermodynamic properties and is only weakly dependent on the optical properties of the metal. The established periodicity after tens of pulses was found to be well below the surface plasmon wavelength, as the observed period is rather attributed to the involvement of evanescent quasi-cylindrical waves [41]. These waves significantly contribute to absorption near individual nanoroughness forming Hertzian dipoles on the surface, reducing the resonant wavelength from near-wavelength *λ* to a sub-wavelength scale ≈3λ/4 via collective dipole–dipole coupling. This way, a feedback effect during the structuring process further reduces the period, reinforcing the subwavelength nature of the patterns.

### 4.2 Two scale patterning

Experimental results shown in Figure 2 confirm that LSFL exhibit a clear spatial organization dictated by coherent interaction between surface-scattered waves and the incident field [40]. The laser beam’s spatial coherence enables a structured interference pattern, overcoming initial surface roughness randomness and producing a well-defined periodic fingerprint [2], [42]. The phase transitions induced by localized energy modulation drive material redistribution, where molten regions reorganize and shape the emerging structures [50]. The multi-pulse regime amplifies these effects through a self-reinforcing mechanism, where curvature-dependent absorption enhances localized energy deposition. The deepening of valleys and the elevation of ridges are governed by feedback loops that accentuate structural contrast. Optical coupling between adjacent features modulates local field intensities, strongly depending on polarization states, resembling near-field Mie scattering effects [41]. This suggests that structuring dynamics integrates polarization-dependent interactions at sub-wavelength scales, as illustrated in Figure 3, even when material-driven pattern formation plays a role.

HSFL formation, while linked to similar physical mechanisms whatever the polarization configurations, exhibits directionality due to local symmetry breaking in the scattered near-field. The anisotropic feature of near-field enhancements, even under circular polarization, emphasizes the role of localized thermal gradients in the orientation of the structure at the nanoscale. The preferential arrangement of these structures along closely spaced protrusions (pillars) suggests a thermally driven organization, reinforcing the influence of temperature-dependent material flow. Figure 3 highlights the contrast between HSFL formed under circular polarization, revealing a locally concentric arrangement along the radial directions, and those formed under linearly polarized pulses, which show a distinct preferential alignment.

A key comparison in Figures 3 and 4 examines the structures generated under circular polarization and the sum of sequentially rotated linear polarizations. Although both integrate multiple polarization states over time, the 2D structuring mechanisms differ. The sum of discrete linear polarizations results in an apparent isotropic distribution but lacks the spatial coherence of pure circular polarization, where interference effects remain consistently phase-matched within the pulse duration. The observation that HSFL remain isotropic under circular polarization but preferentially orient under summed linear polarization, as shown in Figure 3(g) and (n), highlights the initiating role of the polarization state in the formation of HSFL. In particular, scattered waves originating from distinct pillars interfere constructively at subwavelength scales, producing localized field anisotropies, as shown in the highlighted region of Figure 3(b), where three LSFL domains form a trihedral junction and HSFL filaments converge toward the center of this trimer. These anisotropies, combined with thermally induced directional gradients, lead to a preferential alignment of the HSFL filaments, governed by the spatial distribution of near-field energy, which is defined by the coupling between the polarization state and the structures. This comparison unravels how the dynamics of polarization state affects both spatial coherence at the wavelength scale and non-radiative fields distribution around nanoscale protrusions.

LSFLs form a global neural network-like pattern throughout the laser spot, defining a collective behavior due to the spatial coherence of the beam. With circular polarization, the structures often exhibit a local three-fold symmetry (hexagonal networks as observed in Figure 3(f)) at short range, which rotates from one pillar to another, losing long-range order across the impact area. With rotated linear polarizations, at long range, the pattern resembles circular polarization, but local stripes become more prominent, forming a two-fold symmetry at short range and creating a more rectangular grid as revealed by Figure 3(m). In both cases, deformation at the edge of the impact site further rounds the structures as one moves away from the center, while transiently formed surface irregularities locally distort the network. In contrast, HSFL adopt a more localized organization at a scale below the laser wavelength, connecting nearest neighbors through frozen liquid bridges. These filaments lack large-scale order and their organization remains constrained by LSFL-driven curvatures, preventing long-range coherence, in particular for the circular case. However, the potential for 2D self-organization at the nanoscale across the entire spot remains, provided that structures with periodicities near the laser wavelength are avoided and that the surface state is free from large asperities.

## 5 Conclusions

The formation of 2D-LIPSS on various metals under circular polarization reveals a self-organization mechanism that differs fundamentally from structures formed under linear polarization. Unlike the directional anisotropies induced by linear polarization, circular polarization facilitates isotropic integration of scattered waves, resulting in a more homogeneous and coherent structuring process. Experimental observations confirm that LSFL patterns adhere to coherence effects across the laser spot, while HSFLs emerge through local topography-driven optical near-field enhancements, which drive thermal gradients and material redistribution along pre-formed pillars. Comparing circular polarization to the sum of rotating linear polarizations underscores the critical role of feedback, particularly from the final pulses, influenced by both the existing bumpy topography and the final polarization state. Circular polarization ensures greater isotropy, and its ability to generate globally isotropic structures with sub-wavelength periodicity offers a robust approach for advanced laser-driven nanostructuring. At last, HSFL formation provides rich insights, as it serves as a signature of the near fields induced by the final pulses and appears to lose global coherence across the laser spot, instead of being influenced by the interaction with adjacent topographic features formed by LSFL. These findings expand the understanding of ultrafast light–matter interactions and open new avenues for fabricating functional surfaces with custom-designed, isotropic, pillar-like structures.
